# On straightness measurements of large CNC machine tools

**DOI:** 10.1038/s41598-024-63909-9

**Published:** 2024-06-17

**Authors:** Ahmed ElMelegy, Sarwat Zahwi, Ahmed Sobhy

**Affiliations:** Engineering and Surface Metrology Laboratory, National Institute of Standards (NIS), Giza, Egypt

**Keywords:** Large capacity CNC machines, Autocollimators, Production metrology, Straightness errors, Engineering, Nanoscience and technology, Physics

## Abstract

The CNC (computerized numerically controlled) machines are widespread in use due to their high capability of precise manufacturing in industrial production. They have a wide range of designs depending on the working capacity in manufacturing. The associated form errors in large-capacity CNC machines during production shall be identified and corrected or eliminated. This study presents an investigation of one of the main form errors that may affect the manufacturing precision of these machines. This error type is a straightness error with both two kinds of horizontal and vertical errors. The study is carried out for a vertical turning center CNC machine type. The straightness errors are measured for the X axis at different latches in the Z direction and for the Z axis at three positions in the X direction with multi-displacement steps. Different algorithms are used in the determination of straightness errors. The X-axis has minimum horizontal straightness errors at latch Nr. 3 and minimum vertical straightness errors at latch Nr. 5. For the Z axis, the minimum values for horizontal and vertical straightness errors exist when the spindle is located 1200 mm away from the machining center to the right. The displacement steps have a significant impact on the determination of straightness errors.

## Introduction

**Figure 1:**
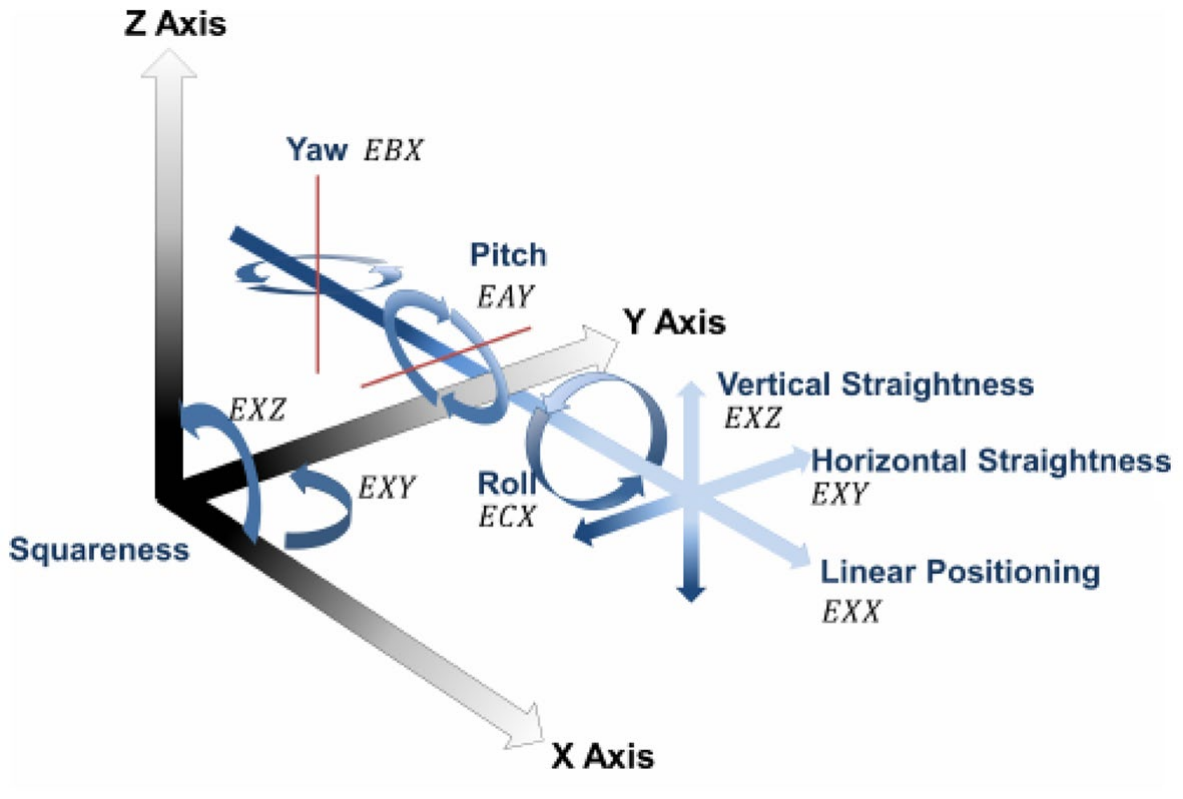
Errors in machine axes.

Metrology and Industrial Production have a clear relationship with each other. The continuous improvement in the quality level of industrial production especially for products of fine and small tolerances requires subsequently metrological capability with sufficient precision and accuracy. To fulfill these demands, the manufacturing tolerances of products and measurement errors of corresponding production machines shall be measured and identified^1^. Computerized numerically controlled (CNC) machines are considered the most powerful tool for industrial production. The precision level of these machines acts as the machining foundation to obtain high precision and accuracy in production processes^2^. CNC machines have different errors that may affect their performance i.e. static, quasi-static, and dynamic errors. These errors especially static ones should be precisely measured to be eliminated or compensated in the manufacturing of products, Fig. 1.

Positioning errors (linear and angle), angular errors (yaw, pitch, and roll), and straightness and squareness errors are clear examples of static errors. Straightness errors are considered one of the important errors that directly affect production machines and their products^3,4^. These errors have two components, horizontal and vertical straightness errors. These two types of straightness errors express the straightness errors in the horizontal and vertical planes during the motion of machines' axes. These errors can result in a direct effect on the straightness and flatness errors of the manufactured products by these machines. The error values of machine straightness should be identified, determined, and minimized^5^. A B-spline curves modeling is used as a base for straightness error determination in large CNC milling machines^5^. One of the proposed methods to determine straightness errors based on the angular errors i.e. yaw and pitch errors is studied in^6^. The straightness error measurement is studied by identifying its relation with the angular errors i.e. pitch, yaw, and roll errors^6,8^. There are different measuring instruments and tools that are used for the measuring of straightness errors i.e. straight edge, ball bar, laser interferometer, autocollimator …. etc. The calibration and traceability of such instruments are necessary to recognize traceability in machine tools measurements^11^. Laser interferometers are used for straightness errors for machines' axes, moving stables, and guideways^9,10^. These interferometers act also as highly precise and fast measuring tools for parallelism and dynamic errors measurements for CNC machines^7,12,13^. Autocollimators are considered the most efficient and versatile instruments for measurements of straightness errors^3,13^. It can measure errors of horizontal and vertical type at the same time with the same setup without any change^3^. For other tools, a lot of time is consumed due to the tool adjusting to measure horizontal straightness errors and readjusting the tool once more to measure vertical ones. Using of autocollimators has additional advantage which is its angular accuracy upto 0.05 arcs. This accuracy cannot be achieved by any other tool. The most important thing that limit this use of autocollimator is the weight of its reflecting mirrors which need good and sufficient fastening to the machine moving parts during measurements. The straightness measurements of CNC machines depend on many factors that may affect the measured errors^9,10^. The location of measurements inside the machine volume as well as measurement steps can directly affect measured values of straightness errors. The analysis algorithm for measurement results also changes the error values^11,12^. The least-square, zero endpoint, and minimum zone are three common analysis tools that are used in error evaluation. Through the literature review for previous research work, two or three gap points exist. Studying of affecting factors on straightness measurements of machine axes and the use of autocollimators in this application. In this study, the straightness measurements of large capacity CNC machine of vertical turning center type are carried out. This machine has three axes, X, Z, and one rotation axis of C^15–18^. It has 6 latches in the vertical direction with a mechanical mechanism carrying the machine spindle and lateral carriage. This mechanism can be repositioned from one latch to another according to the size of the manufactured part. The straightness errors are measured on the X- axis at different latches and on the Z-axis at different positions inside the machine volume. The measured errors are analyzed in a comparative way by different algorithms. In the next section, a description of the measuring instrument, machine type, and measurement procedure are explained. In Section “Results”, the measurement results are represented in numerical and graphical shapes. In Section “Uncertainty evaluation”, the results are discussed at different measurement conditions. In Section “Discussion”, the main conclusions are outlined. The measurements that are carried out in this study are related to no-load or quasi-static measurements of machine tools^14^. The straightness measurements are measured in two axes X and Z one after one, not at the same time.

## Material and methods

In this study, an autocollimator system is used for measurement and determination of straightness errors of large capacity CNC machine^3^. The measuring instruments; autocollimator and CNC machine are illustrated in the detailed description in Sections “The autocollimator system” and “CNC machine tool”. The experimental set as well as the measurement method are explained in Section “Measurement method”.

### The autocollimator system

**Figure 2:**
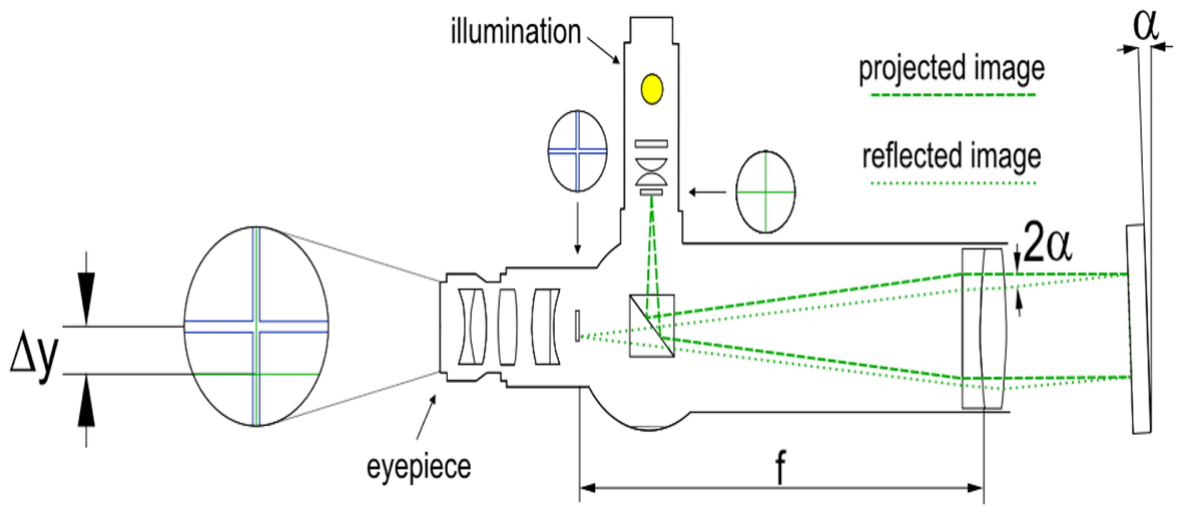
Measurement principle by autocollimator.

An Electronic autocollimator instrument of Elcomat 3000 model type (Moeller-Wedel—Germany) is used. The instrument is capable of measuring angular deviations within a range of ± 1000 arcs with an accuracy of 0.05 arcs. In general, Autocollimators can measure of smallest inclination deviation in two perpendicular axes in fractions of arc-second (arcs)^13^, Fig. 2.

**Figure 3:**
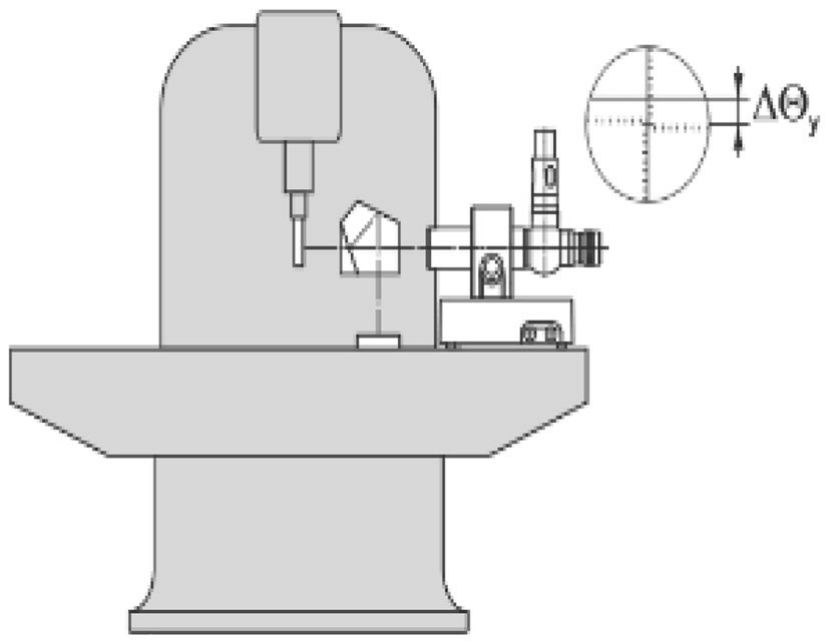
Straightness measurements by autocollimator system^13^.

The image of a located illuminated object in the rear focal plane of the collimator lens is projected to infinity while it is reflected by a mirror. A light-sensitive receiver picks up the image. A slight tilt of the angle between the optical axis of both the autocollimator and mirror causes a deviation that can be precisely detected. Autocollimators are mainly used for measurements of angular errors (yaw and pitch), parallelism, straightness, and squareness errors, Fig. 3.

### CNC machine tool

**Figure 4:**
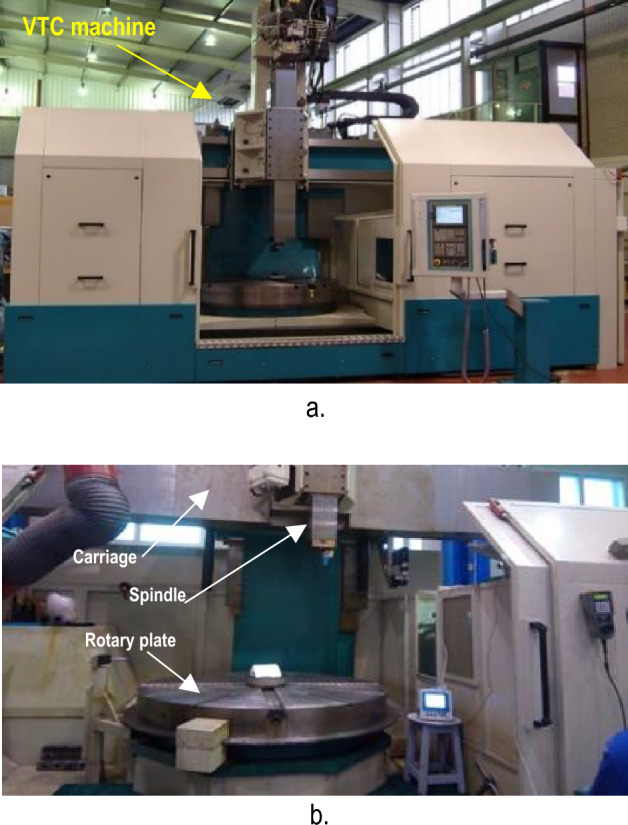
Vertical turning centering CNC machine (**a**, **b**)^3^.

**Figure 5:**
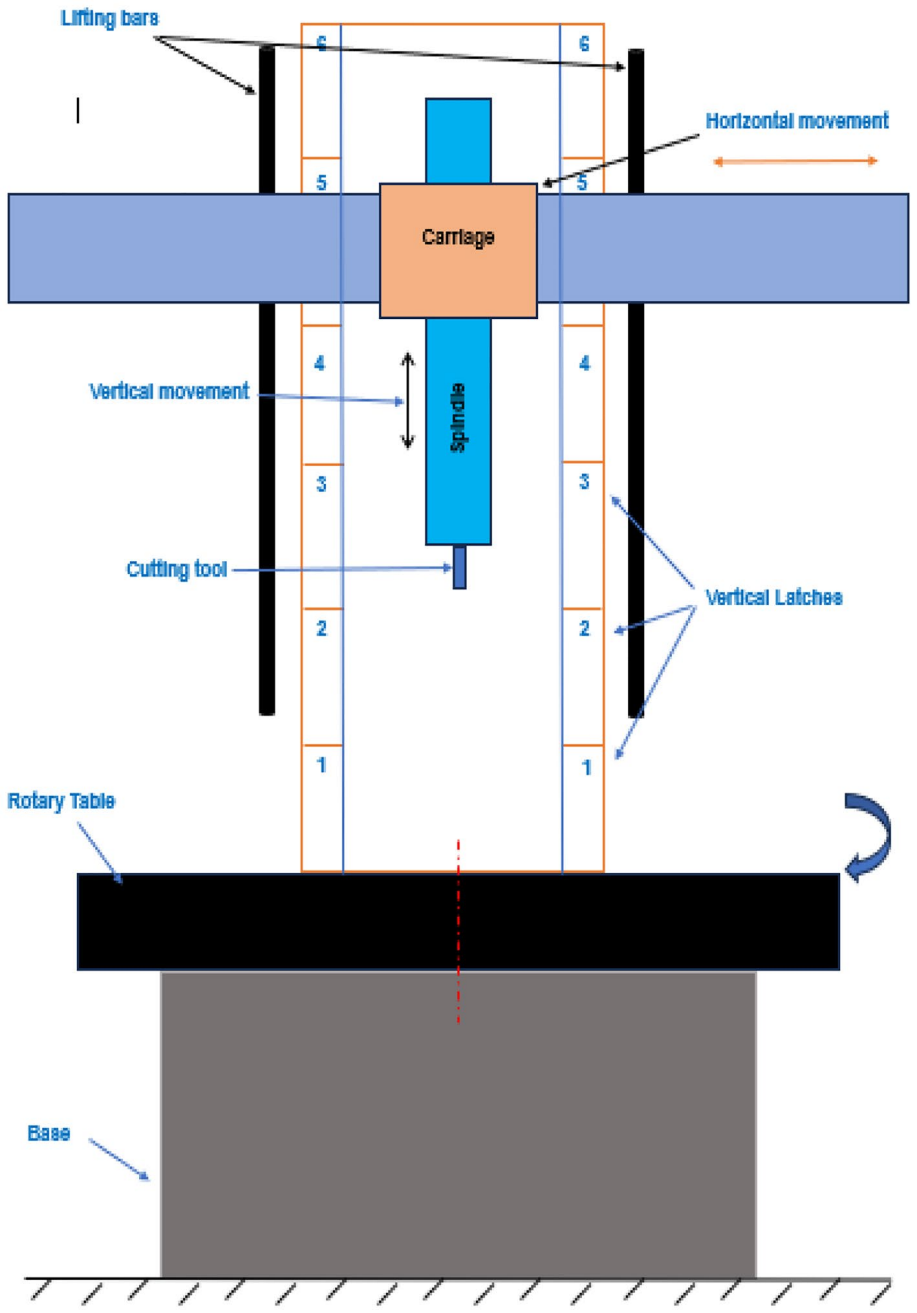
Schematic drawing of VTC machine.

In this work, the measurements of straightness errors are carried out for large capacity CNC machine type. It is a vertical turning-center (VTC) machine of Webster Bennett (WB) Evolution Model. The machine has three axes; two linear axes of X axis (horizontal) and Z axis (vertical) and C axis (rotation). The C axis is a rotary table that can rotate precisely a complete circle (360°) with accuracy up to 1 min, Fig. 4. This machine has a mechanical mechanism that carries the spindle and lateral carriage and can be repositioned at 6 latches in the vertical direction starting from below according to the size of the manufactured part. The machine spindle can move in X and Z axes for distances of 3000 mm and 1500 mm respectively, Fig. 5. The 6 latches increase the volumetric capacity of the machine in the vertical direction for about an additional 3000 mm (500 mm/latch).

### Measurement method

The straightness error measurements are performed at discrete positions in different axes. These measurements are carried out at different locations inside the machine volume in both X and Z axes and at different measurement steps. The experimental setup is performed through the alignment of the autocollimator head with a reflecting mirror that is fixed to the machine spindle one time in the X axis and another time in the Z axes, Fig. 4a and b. The CNC machine is programmed to move in X and Z axes according to the codes:

**Table:** 

For X axis:	For Z axis:
G91 G54	G91 G54
G1 X = 0 Z = 0 F500	G1 X = 0 Z = 0 F500
X = − 400	Z = 200
M30	M30
= eof =	= eof =

The straightness errors are also analyzed comparatively by different algorithms; regression, end-point fitting, and ISO 1101 (exact feature).

## Results

### Different location positions

#### X-axis

**Figure 6:**
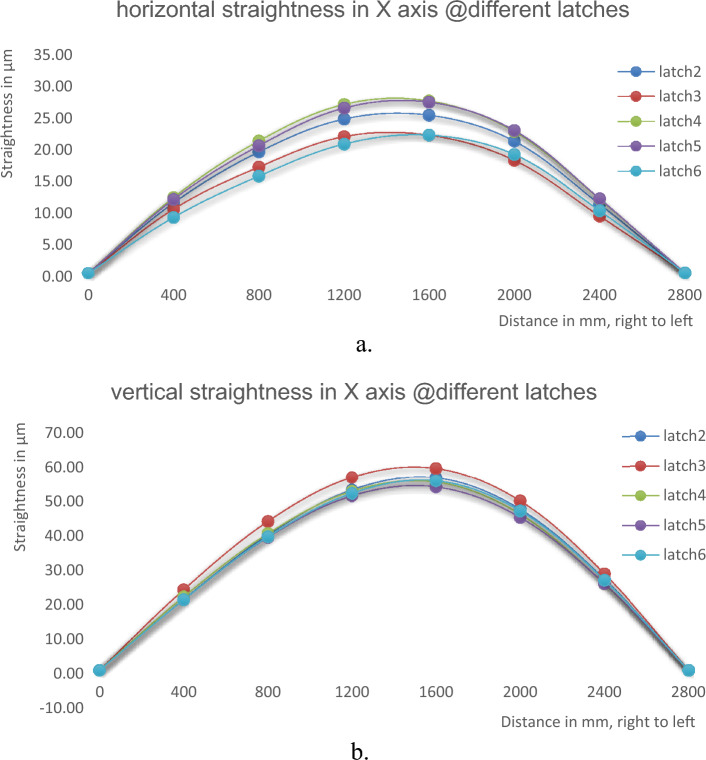
Straightness errors in X axis at different latches; (**a**) horizontal straightness, (**b**) vertical straightness.

The straightness errors are measured on the X-axis at different latches in vertical direction. Each time the mechanical setup that carries the machine spindle and lateral carriage moves vertically from one latch to another, the autocollimator is re-aligned with the fixed mirror to the machine spindle, and the measurements are started. Due to some difficulties, the mechanical setup moves only between latches 2, 3, 4, 5, and 6. The horizontal and vertical straightness errors in the X-axis at different latches are shown in Tables 1 and 2; and Fig. 6a and b.

**Table 1:** Horizontal straightness errors in X axis at different latches.

Latch Nr.	2	3	4	5	6
Steps in mm	Horizontal straightness errors, µm				
0	0.00	0.00	0.00	0.00	0.00
400	11.09	10.11	11.95	11.65	8.81
800	19.12	16.75	20.85	20.10	15.32
1200	24.30	21.53	26.61	26.03	20.36
1600	24.91	21.76	27.16	26.96	21.78
2000	20.83	17.80	22.35	22.52	18.72
2400	10.88	9.02	11.68	11.76	9.86
2800	0.00	0.00	0.00	0.00	0.00
Total straightness errors (TSE), µm	24.91	21.76	27.16	26.96	21.78

**Table 2:** Vertical straightness errors in X axis at different latches.

Latch Nr	2	3	4	5	6
Steps in mm	Vertical straightness errors, µm				
0	0.00	0.00	0.00	0.00	0.00
400	20.57	23.42	21.37	20.49	20.51
800	39.33	43.27	39.76	38.66	38.81
1200	52.48	56.14	52.06	50.72	51.45
1600	56.02	58.78	54.79	53.33	55.14
2000	46.82	49.25	45.38	44.44	46.31
2400	26.47	28.06	25.08	25.18	26.21
2800	0.00	0.00	0.00	0.00	0.00
Total straightness errors (TSE), µm	56.02	58.78	54.79	53.33	55.14

#### Z axis

**Figure 7:**
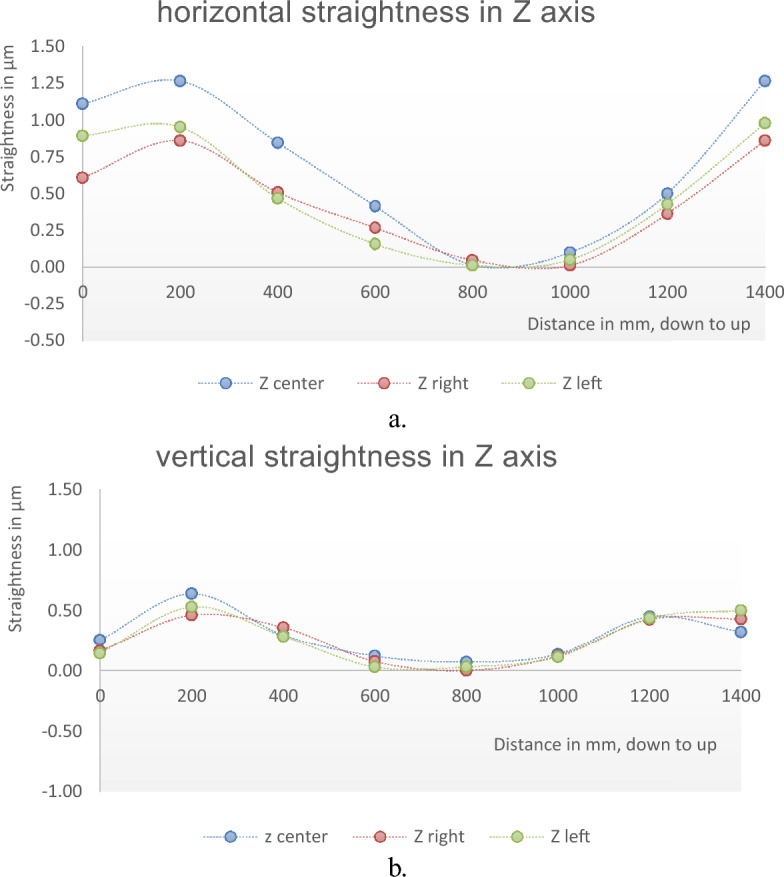
Straightness errors in Z axis at different location in machine volume; (**a**) horizontal straightness, (**b**) vertical straightness.

The straightness errors are measured in the Z axis at different locations in machine volume. The machine has a displacement range of 2500 mm (the machine reads it 5000 Ømm) in the X-axis. The machine spindle is sited at three locations in the X axis, machine center, 1250 mm (2500 Ømm) to the right, and 1250 mm (2500 Ømm) to the left. At each location the autocollimator setup is changed and aligned with the fixed reflecting mirror to the machine spindle and measurements are performed. The measurement results for straightness errors in the Z-axis at these three locations are shown in Tables 3 and 4; and Fig. 7a and b.

**Table 3:** Horizontal straightness errors in Z axis at different lateral positions.

Position	Center	1250 mm right	1250 mm left
Steps in mm	Horizontal straightness errors, µm		
0	1.11	0.61	0.89
200	1.27	0.86	0.95
400	0.85	0.51	0.47
600	0.42	0.27	0.16
800	0.02	0.05	0.01
1000	0.10	0.01	0.05
1200	0.50	0.36	0.43
1400	1.27	0.86	0.98
Total straightness errors (TSE), µm	1.27	0.86	0.98

**Table 4:** Vertical straightness errors in Z axis at different lateral positions.

Position	Center	1250 mm right	1250 mm left
Steps in mm	Vertical straightness errors, µm		
0	0.26	0.16	0.14
200	0.64	0.46	0.53
400	0.30	0.36	0.29
600	0.12	0.08	0.03
800	0.07	0.00	0.03
1000	0.14	0.12	0.11
1200	0.45	0.43	0.44
1400	0.32	0.43	0.50
Total straightness errors (TSE), µm	0.64	0.46	0.53

### Different measurement steps

**Figure 8:**
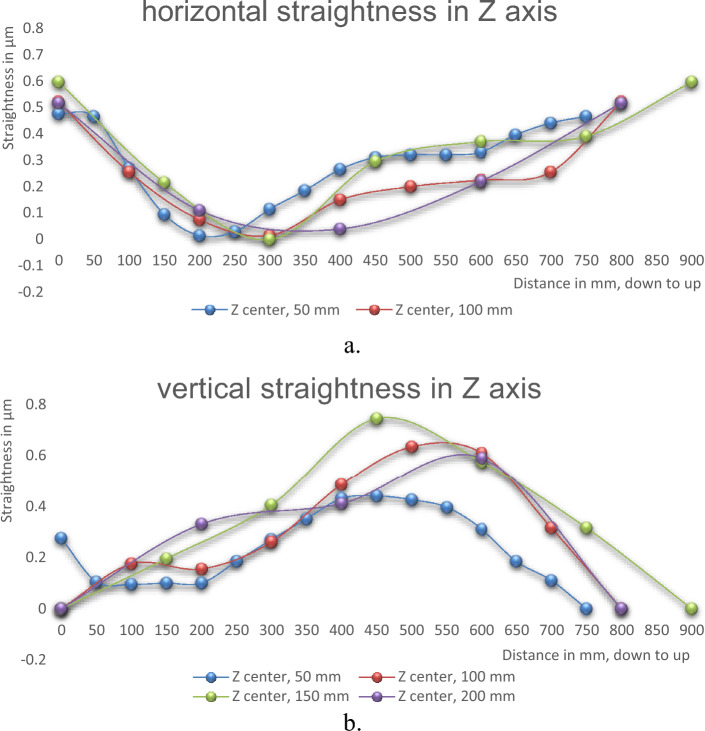
Straightness errors in Z axis at different measurement steps, (**a**) horizontal straightness, (**b**) vertical straightness.

The measurements of straightness errors are also carried out at different measurement steps. The measurements are done in the Z axis where the spindle is located at the center of the machine. Four series of measurements in steps of 50, 100, 150, and 200 mm are performed. In each run, the measurement step is changed. The measurements are carried out for a total displacement of about 750 mm. The measured straightness errors at different measurement runs are presented in Tables 5 and 6; and Fig. 8a and b.

**Table 5:** Horizontal straightness errors in Z axis at different measurement steps.

Steps	50 mm	100 mm	150 mm	200 mm
Points in mm	Straightness errors, µm			
0	0.475	0.52	0.60	0.52
50	0.465	–	–	–
100	0.27	0.26	–	–
150	0.095	–	0.22	–
200	0.015	0.08	–	0.11
250	0.03	–	–	–
300	0.115	0.02	0.00	–
350	0.185	–	–	–
400	0.265	0.15	–	0.04
450	0.31	–	0.30	–
500	0.32	0.20	–	–
550	0.32	–	–	–
600	0.33	0.23	0.37	0.22
650	0.395	–	–	–
700	0.44	0.26	–	–
750	0.465	–	0.39	–
800	–	0.52	–	0.52
850	–	–	–	–
900	–	–	0.60	–
Total straightness errors (TSE), µm	0.475	0.52	0.60	0.52

**Table 6:** Vertical straightness errors in Z axis at different measurement steps.

Steps	50 mm	100 mm	150 mm	200 mm
Points in mm	Straightness errors, µm			
0	0.275	0.00	0.00	0.00
50	0.105	–	–	–
100	0.095	0.18	–	–
150	0.1	–	0.20	–
200	0.1	0.16	–	0.33
250	0.185	–	–	–
300	0.27	0.26	0.41	–
350	0.35	–	–	–
400	0.43	0.49	–	0.41
450	0.44	–	0.75	–
500	0.425	0.64	–	–
550	0.395	–	–	–
600	0.31	0.61	0.58	0.59
650	0.185	–	–	–
700	0.11	0.32	–	–
750	0	–	0.32	–
800	–	0.00	–	0.00
850	–	–	–	–
900	–	–	0.00	–
Total straightness errors (TSE), µm	0.44	0.64	0.75	0.59

### Different analysis tools

**Figure 9:**
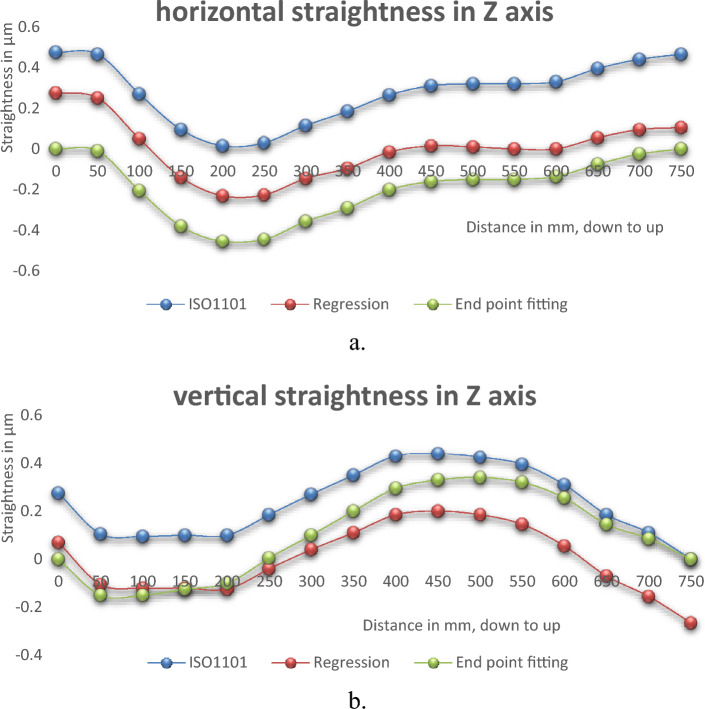
Straightness errors with different analysis method. (**a**) horizontal straightness, (**b**) vertical straightness.

Different tools are used to analyze the measured straightness errors. These tools are Regression, End-Point Fitting, and ISO 1101 (exact feature). The straightness errors are measured for 750 mm range in the Z axis at the center position with moving step 50 mm. The determinations of straightness errors are presented in Tables 7 and 8 and Fig. 9a and b.

**Table 7:** Horizontal straightness errors in Z axis by different analysis tools.

Analysis tool	ISO1101	Regression	End point fitting
Points in mm	Straightness errors, µm		
0	0.475	0.275	0.000
50	0.465	0.250	− 0.010
100	0.27	0.050	− 0.205
150	0.095	− 0.140	− 0.380
200	0.015	− 0.230	− 0.455
250	0.03	− 0.225	− 0.445
300	0.115	− 0.145	− 0.355
350	0.185	− 0.095	− 0.290
400	0.265	− 0.015	− 0.200
450	0.31	0.015	− 0.160
500	0.32	0.010	− 0.150
550	0.32	0.000	− 0.150
600	0.33	0.000	− 0.135
650	0.395	0.055	− 0.075
700	0.44	0.095	− 0.025
750	0.465	0.105	0.000
Total straightness errors (TSE), µm	0.475	0.505	0.455

**Table 8:** Vertical straightness errors in Z axis by different analysis tools.

Analysis tool	ISO1101	Regression	End point fitting
Points in mm	Straightness errors, µm		
0	0.275	0.070	0.000
50	0.105	− 0.105	− 0.150
100	0.095	− 0.120	− 0.150
150	0.1	− 0.120	− 0.125
200	0.1	− 0.125	− 0.100
250	0.185	− 0.040	0.005
300	0.27	0.040	0.100
350	0.35	0.110	0.200
400	0.43	0.185	0.295
450	0.44	0.200	0.330
500	0.425	0.185	0.340
550	0.395	0.145	0.320
600	0.31	0.055	0.255
650	0.185	− 0.070	0.145
700	0.11	− 0.155	0.085
750	0	− 0.265	0.000
Total straightness errors (TSE), µm	0.44	0.465	0.490

## Uncertainty evaluation

In this work; straightness errors are measured at several conditions; different machine positions in the X and Z axes, different travel steps, and different analysis tools. To obtain accurate measurement, the associated uncertainties should be evaluated^10^. The affecting factors on the measurement process should be determined. The uncertainties due to *repeatability, instrument calibration, resolution, optical miss alignment, and temperature effect* should be considered for such types of measurements. The evaluated uncertainties in other conditions are presented in tables Table 10. The straightness errors can be determined according to;

1 $$ S_{E} = \, \Delta \, + \varepsilon_{R} $$

where; S_E_ is the straightness error, Δ = (maximum error − minimum error) along the measured length and ε_R_ is residual errors due to instrument calibration, resolution, optical miss alignment, and temperature effect.

Assuming a linear model and sensitivity coefficients equal 1. by differentiation of Eq. (1); the contributory variances are

2 $$ u^{2} \left( {S_{E} } \right) \, = \, u^{2} \left( \Delta \right) + u^{2} \left( {\varepsilon_{1} } \right) + \, u^{2} \left( {\varepsilon_{2} } \right) + u^{2} \left( {\varepsilon_{3} } \right) + u^{2} \left( {\varepsilon_{4} } \right) $$

where; u(S_E_) uncertainty in straightness errors, u(Δ) uncertainty for measurements' repeatability, u(ε_1_) uncertainty for instrument calibration, u(ε_2_) uncertainty for instrument resolution, u(ε_3_) uncertainty for optical misalignment, u(ε_4_) uncertainty for temperature effect.

The associated calibration uncertainty will be;

3 $$ u\left( {S_{E} } \right) \, = \, \left[ {u^{2} \left( \Delta \right) + u^{2} \left( {\varepsilon_{1} } \right) + u^{2} \left( {\varepsilon_{2} } \right) + u^{2} \left( {\varepsilon_{3} } \right) + u^{2} \left( {\varepsilon_{4} } \right)} \right]^{1/2} $$

the expanded uncertainty of calibration can be determined by;

4 $$ U\left( {S_{E} } \right) \, = \, K \cdot u\left( {S_{E} } \right) $$

where; K is a coverage factor related to the confidence level. It depends on all contributors' effective degree of freedom and the number of repetitions of measurement results.

In Table 9, the uncertainty in straightness errors in X axis at latch 2 will be clearly described.

**Table 9:** Evaluation of uncertainty in the determination of straightness errors in X axis at latch 2.

Uncertainty sources	Standard uncertainty	Distribution	Degree of freedom
Repeatability	0.63 µm	Normal	9
Instrument calibration	0.03 µm	Normal	∞
Instrument resolution	0.03 µm	Rectangular	∞
Optical misalignment	0.03 µm	Rectangular	∞
Environmental conditions	0.03 µm	Rectangular	∞
Combined uncertainty (u)	0.64 µm	Normal distribution, effective degree of freedom = ∞	
Expanded uncertainty (U)	1.28 µm	At coverage factor K = 2 & confidence level 95%	

## Discussion

In this work, the straightness errors are determined at different locations in the machine volume. The machine operator should know exactly the mapping errors for the machine that he deals with^14^. Some researchers are interested in the explanation of measurement techniques^6,9^, and the comparison of results of straightness measurements that are obtained by different instruments^3^. The point of research on the location effect on the straightness measurements has been uncovered before. The authors claim that the other two research points; displacement steps and analysis tools still need to be studied in this field.

### Different location positions

**Figure 10:**
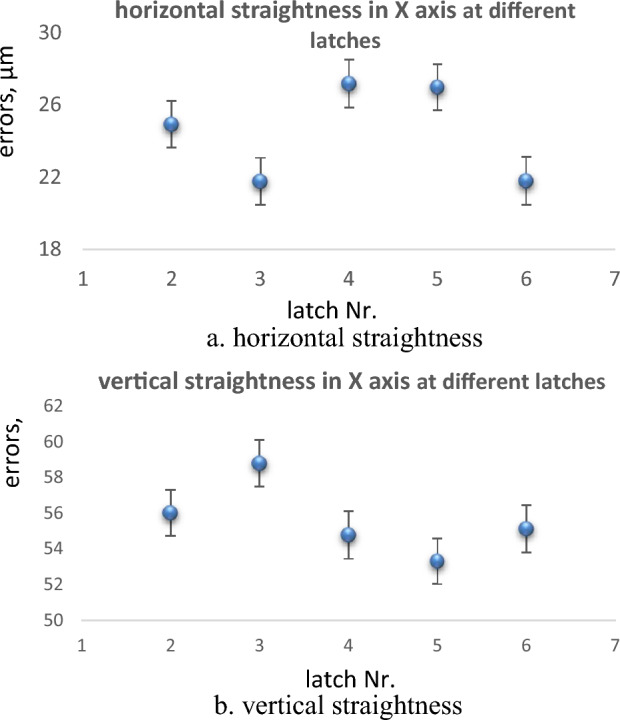
Straightness errors in X axis at different latches; (**a**) horizontal straightness, (**b**) vertical straightness.

The straightness errors are measured in the X axis for the 2800 mm displacement range which acts about 90% of the full range of the X axis (3000 mm). As shown in Fig. 6, the straightness errors in the X-axis are measured at latches 2, 3, 4, 5, and 6. Figure 6a shows the horizontal straightness errors while Fig. 6b. shows the vertical straightness errors that are measured at each latch. The straightness curves have approximately similar shapes in all latches. The value of total errors (TSE) of horizontal straightness ranges from 21.76 µm at latch 3 (minimum) to 27.16 µm at latch 4 (maximum). For vertical straightness, TSE ranges from 53.33 µm at latch 5 to 58.78 µm at latch 3. The associated uncertainties for measurements at different latches are evaluated as in Table 10. The obtained results for TSE with their uncertainties are intersected by each other at different latches, Fig. 10a and b. This indicates that the results for horizontal straightness are consistent. The machine operator should determine in which latch, the machine should be sited.

**Table 10:** Evaluation of uncertainty in determination of straightness errors at different conditions.

Measurement conditions	Expanded uncertainty	Measurement conditions	Expanded uncertainty
In X axis, latch 3	1.30 µm	Step 50 mm	0.12 µm
In X axis, latch 4	1.33 µm	Step 100 mm	0.11 µm
In X axis, latch 5	1.27 µm	Step 150 mm	0.11 µm
In X axis, latch 6	1.32 µm	Step 200 mm	0.10 µm
In Z axis, center	0.15 µm	ISO1101	0.11 µm
In Z axis, left	0.16 µm	Regression	0.10 µm
In Z axis, right	0.13 µm	End-point fitting	0.12 µm

**Figure 11:**
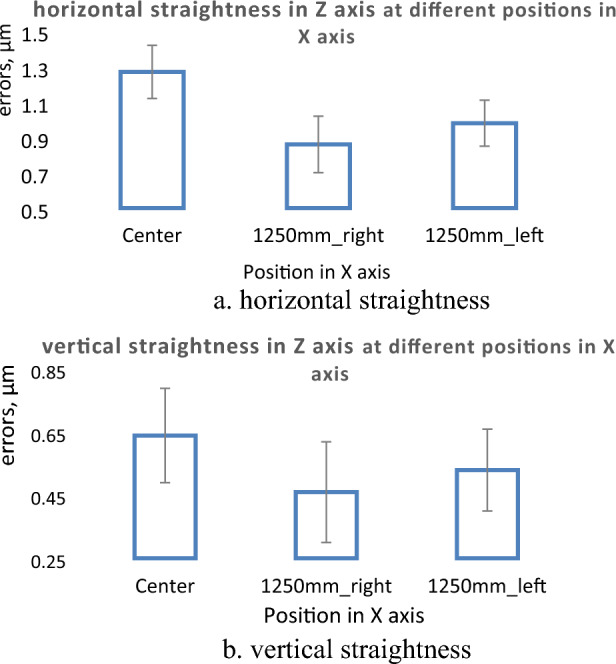
Straightness errors in Z axis at different locations in machine volume; (**a**) horizontal straightness, (**b**) vertical straightness.

For straightness measurements in the Z axis, the measurements are carried out for the 1400 mm displacement range (~ 93% of the Z axis displacement range). The straightness errors are measured at three positions, machine center, 1250 mm to right, and 1250 mm to left, Fig. 7a and b. The error curves have sinusoidal shapes with maximum and minimum values of 1.27 µm and 0 µm at 200 mm and 900 mm respectively for horizontal straightness. For vertical straightness, the maximum and minimum values are 0.64 µm and 0 µm at 200 mm and 800 mm respectively. There is no observed inconsistency between the results for either horizontal or vertical straightness at different positions in the Z axis, Fig. 11a and b. It is advised to set the machine at 1250 mm to the right during the machining in the Z axis.

### Different displacement steps

**Figure 12:**
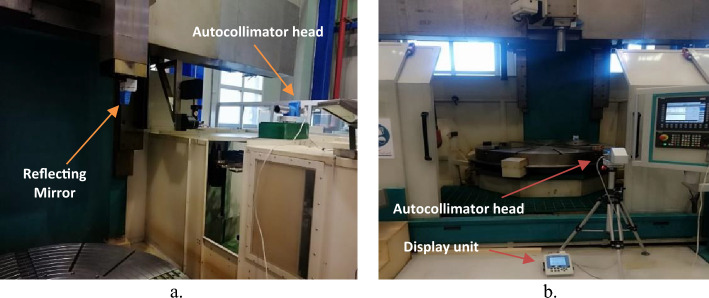
Measurements of straightness errors by autocollimator system in, (**a**) X axis and (**b**) Z axis^3^.

**Figure 13:**
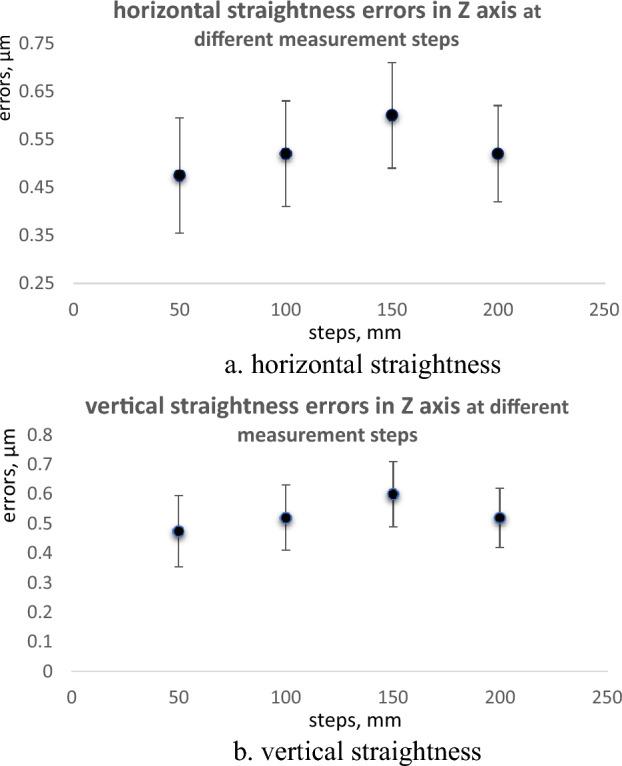
Straightness errors in Z axes at different measurement steps. (**a**) horizontal straightness, (**b**) vertical straightness.

The measurements of straightness are done at multi-displacement steps of 50 mm, 100 mm, 150 mm, and 200 mm. The straightness measurements are carried out in the Z axis at the machining center for a displacement range of 750 mm, Figs. 5 and 12. For horizontal straightness, the values of total errors (TSE) range from 0.475 µm (minimum) at 50 mm step to 0.60 µm (maximum) at 150 mm step. For vertical straightness, the total errors (TSE) varied from 0.44 µm (minimum) at 50 mm step to 0.75 µm (maximum) at 150 mm step. The obtained results for TSE with their uncertainties are intersected by each other at different latches, Fig. 13a and b. In general, the straightness measurements with the step of 50 mm have minimum values in straightness errors. The determination of the displacement step during the straightness measurements is necessary to get a real picture of the straightness errors of the machine and avoid any inherent sources that may affect these measurements.

### Different analysis tools

**Figure 14:**
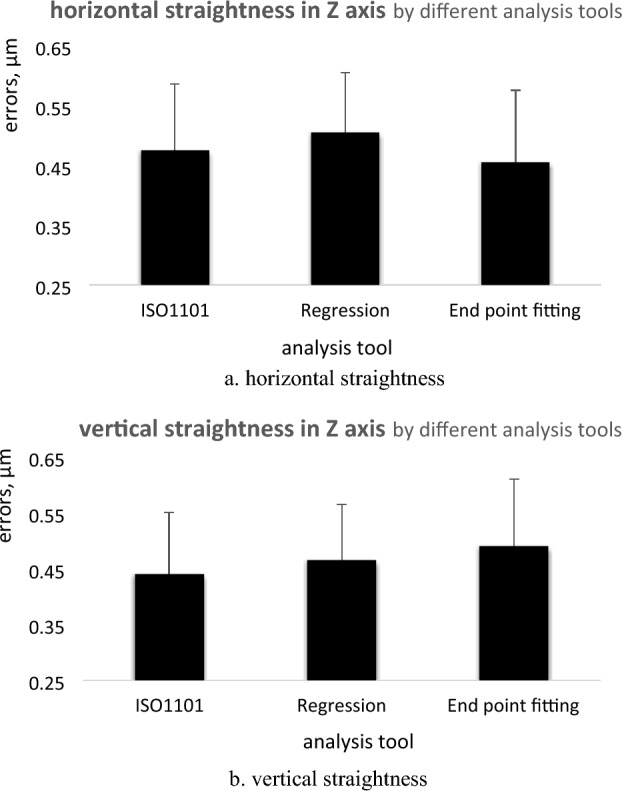
Straightness errors in different analysis methods; (**a**) horizontal straightness, (**b**) vertical straightness.

The straightness errors are determined by different analysis tools. Methods of ISO1101 (exact feature), endpoint fitting, and regression are used. Different error determinations are performed for straightness measurements in the Z axis at the machining center with a displacement step of 50 mm, Fig. 9a and b. the analysed results by different tools are consistent with each other. There are some differences in straightness error determination of 10% for horizontal straightness measurements and 25% for vertical straightness measurements. It ranges from 0.455 µm for ISO1101 (minimum) to 0.505 µm for regression (maximum) for horizontal errors, Fig. 14a. For vertical straightness, it varies from 0.44 µm by ISO1101 upto 0.49 µm by end fitting, Fig. 14b. For straightness measurements, the analysis method should be known and selected to be appropriate for the measurement type.

## Conclusions

The presented study concerns the determination of straightness errors of vertical turning center CNC machine type as a clear example of large CNC machine tools. The measurement method that is used in this work are based on ISO standard method^14^. In addition to that the measuring instrument are traceable to SI units. These two items ensure the validity of presented results and measurements. This work aims to study different affecting factors to give a real picture of the straightness errors of the machine tool. Three factors are studied; different vertical and horizontal axes positions, multi-displacement intervals, and analysis methods. The X axis has minimum values of horizontal straightness errors when the mechanical setup is located at latch no. 3 and minimum values of vertical straightness errors at latch no. 5. The machine operator should determine in which latch, the machine should be sited. For the Z axis, the minimum values for horizontal and vertical straightness errors existed when the spindle was located 1200 mm away from the machining center to the right. It is advised to set the machine at 1250 mm to the right during the machining in the Z axis. The minimum errors for horizontal and vertical straightness at different displacement steps are found at the step of 50 mm. The difference in error determinations by the analysis tools of ISO1101 (exact feature), endpoint fitting, and regression methods; ranges from 10 to 25%, and in general; error determination by ISO1101 has minimum values. The machine operator can make corrections due to the presented machine errors in the operating software or if it is not possible, he has to choose positions with minimum straightness errors to locate the mechanical setup in the manufacturing of products. For the future; the authors think about comparing the presented results with the measurements of straightness errors on workpieces that are manufactured by the machine used in this study.

## Data Availability

All data generated or analyzed during this study are included in this paper.

## Abbreviations

CNC: Computerized numerical controlled
CCD camera: Charged-coupled device camera
VTC: Vertical turning-center
